# Deprescribing potential of commonly used medications among community-dwelling older adults: insights from a pharmacist’s geriatric assessment

**DOI:** 10.1038/s41598-024-56780-1

**Published:** 2024-03-14

**Authors:** Iva Bužančić, Margita Držaić, Ingrid Kummer, Maja Ortner Hadžiabdić, Jovana Brkić, Daniela Fialová

**Affiliations:** 1 City Pharmacies Zagreb, Kralja Držislava 6, Zagreb, Croatia; 2 Faculty of Pharmacy and Biochemistry, Center for Applied Pharmacy, University of Zagreb, Ante Kovačića 1, 10 000 Zagreb, Croatia; 3 Department of Social and Clinical Pharmacy, Faculty of Pharmacy in Hradec Králové, Charles University, Akademika Heyrovského 1203/8, Hradec Králové, Prague, Czech Republic; 4 Department of Social Pharmacy and Pharmaceutical Legislation, Faculty of Pharmacy, University of Belgrade, 450 Vojvode Stepe Street, Belgrade, Serbia; 5 Department of Geriatrics and Gerontology, 1st Faculty of Medicine in Prague, Charles University, Kateřinská 32, Prague, Czech Republic

**Keywords:** Deprescribing, Healthy ageing, Geriatrics, Geriatric assessment, Polypharmacy, Health care, Medical research, Risk factors

## Abstract

Pharmacist’s geriatric assessment can provide valuable insights into potential deprescribing targets, while including important information on various health-related domains. Data collected from a geriatric assessment questionnaire, for 388 patients, from the Croatian cohort of the EuroAgeism H2020 ESR 7 international project, along with guideline-based deprescribing criteria, were used to analyse potentially inappropriate prescribing of four medication groups (benzodiazepines (BZN), proton pump inhibitors (PPI), opioids, and non-steroidal anti-inflammatory drugs (NSAID)), and to assess the deprescribing potential. Binary logistic regression was used to explore the effects of age, gender, number of medicines and diagnoses, self-reported health, frailty score, and healthcare utilization on the likelihood of needing deprescribing. More than half of participants (n = 216, 55.2%) are candidates for deprescribing, with 31.1% of PPI, 74.8% of NSAID, 75% of opioid, and 96.1% of BZN users meeting at least one criterion. Most common criteria for deprescribing were inappropriately long use and safety concerns. Women (aOR = 2.58; *p* < 0.001), those reporting poor self-reported health (aOR = 5.14; *p* < 0.001), and those exposed to polypharmacy (aOR = 1.29; *p* < 0.001) had higher odds of needing to have medicines deprescribed. The high rate of deprescribing potential warrants prompt action to increase patient safety and decrease polypharmacy. Pharmacist’s geriatric assessment and deprescribing-focused medication review could be used to lead a personalised approach.

## Introduction

In an aging world, healthy aging is a priority for all stakeholders, including older adults, healthcare providers, policy makers, and social care professionals. Healthy aging is defined as the process of developing and maintaining the functional ability that enables well-being in older age^1^. Use of medication to improve health and increase life expectancy is ubiquitous, especially in older adults, but prescribed medication can in some individuals become potentially inappropriate leading to undesirable outcomes such as adverse drug events, hospitalizations, and increased morbidity and mortality^2–4^.

Commonly prescribed and used medications, such as, proton pump inhibitors (PPI), non-steroidal anti-inflammatory drugs (NSAID), opioid analgesics (OPI), or benzodiazepine receptor agonists (BZN) can be inappropriate for older adults^2,3^. To help reduce the risk of use of potentially inappropriate medications (PIMs) and improve outcomes, patients should be introduced to the concept of deprescribing, the healthcare provider-led process of dose reduction or withdrawal of medication which are no longer of benefit to the patient^5,6^. Deprescribing, as a patient-centred process besides taking into consideration patient-related factors, should encompass comprehensive medication history, identifying of potential targets for deprescribing, prioritising and determining which medication can be ceased, planning and initiating the withdrawal process, and providing follow-up care to the patient^7^. Each step in deprescribing requires adequate information, time, clinical experience and knowledge, as well as patient involvement. Conducting comprehensive geriatric assessment can provide valuable insights into potential deprescribing targets, while also including crucial information from various health-related domains^8,9^. Comprehensive geriatric assessment involves a systematic evaluation of older persons, provided by a team of health professionals, which identifies medical, psychosocial, and functional capabilities in order to develop a coordinated plan to maximize overall health with ageing^10^. The content of the assessment varies depending on settings of care (outpatient, hospital, long term care facilities), and can be completed by different members of a multidisciplinary team. Geriatric syndromes, identified during comprehensive geriatric assessment, are often worsened by medicines. Pharmacists, as a part of a multidisciplinary team, can be well placed to help provide pharmacist’s geriatric assessment, prevent potentially inappropriate prescribing and support deprescribing in the management of geriatric syndromes^11,12^.

The aim of this study is to analyse potentially inappropriate prescribing, and the deprescribing potential of four commonly used medicines (prescription and over-the-counter PPI, prescription and over-the-counter NSAID, and restricted prescription OPI and BZN) among community-dwelling older adults. Additionally, we aimed to explore potential factors associated with increased likelihood of needing to have medicines deprescribed.

## Methods

### Data collection and participants

Data were collected as a part of the EuroAgeism H2020 ESR 7 international project entitled ‘*’Inappropriate prescribing and availability of medication safety and medication management services in older patients in Europe and other countries*”^13^, using a standardized, and piloted 17-part research questionnaire for comprehensive geriatric assessment. Participants’ input, as well as available medical records (medical history, laboratory values), and dispensing data were used to complete the questionnaire. Questionnaire included sociodemographic, clinical, medication-related, and service-use related domains, with core clinical components of comprehensive geriatric assessment examined with questions on nutritional status (Mini Nutritional Assessment-short form^14^), mobility and strength (SARC-F questionnaire^15^), activities of daily living (Activities of Daily Living Hierarchy scale^16^), frailty (Clinical Frailty scale^17^), cognitive status (Cognitive Performance scale^18^), mood (self-reported mood items based on Minimum Data Set-based depression rating scale^19^), self-reported health, falls, pain frequency and control (short-form McGill questionnaire^20^), diagnoses, and symptoms. The choice of scales used in the questionnaire was carefully selected by a multidisciplinary team consisting of clinical pharmacists, gerontologists, geriatricians, and academic researchers with the background in clinical pharmacy and geriatrics. Pharmacist’s geriatric assessment included the use of aforementioned questionnaire and assessment of deprescribing potential described below.

This was an observational, cross-sectional study, conducted in Croatia, from June 2019 to December 2020. Community pharmacists, trained in the use of geriatric clinical scales which comprised the questionnaire, from three geographically different regions (north-western (City of Zagreb) and north-eastern continental (Slavonia), and coastal region (Istria)) approached community-dwelling older adults with the invitation to participate in the project. Pharmacists used convenience sampling when approaching potential participants. Participants were included if they were 65 years or older, of stable health (no palliative or terminal care, no acute worsening of health requiring hospitalization or emergency department visit in the last 3 days, and with life expectancy longer than 1 year), using at least one medication, willing to give informed consent, and without sever communication disorders (unable to speak or hear) or dementia. Pharmacists conducted the interviews, in a separate part of the community pharmacy to ensure privacy and comfort. On average interviews lasted between 45 and 75 min. To avoid participant fatigue, or when medical documentation was needed to support participants’ recall, pharmacist and participant arranged subsequent meetings to complete the questionnaire.

For this analysis, parts of collected data were used: sociodemographic, data on lifestyle (smoking, alcohol intake, diet), frailty score (examined using the clinical frailty scale from “very fit” (1) to “terminally ill” (9)), changes in cognitive status (no changes, improvement or worsening of cognitive status), healthcare utilization (hospitalizations and emergency departments visits within the last 12 months), diagnoses, symptoms (present in the past 7 days), self-reported health score (scale from “very poor” (0), “poor”(1), ”moderate” (2), ”good” (3), ”very good” (4)), pain frequency and control (examined using the short form McGill pain questionnaire and diagram with frequency scale from “multiple times a day” (0), “once daily” (1), “couple of time a week” (2) to “rarely” (3) and numeric pain intensity scale from “no pain” (0) to “worst possible pain” (10)), history of falls, and detailed information on the use of, and adherence to prescription and over-the-counter medicines as well as herbal and dietary supplements.

Sample size was determined based on census data on the number of adults 65 years and older in Croatia, using a single population proportion formula, with a 95% confidence level and relative precision of 5%, and was calculated to 385 participants^21^. This aligned with the EuroAgeism H2020 ESR 7 projects protocol on number of participants from each participating country. Ethical approval was obtained from the Ethical committees of the Charles University (Czech Republic, EuroAgeism H2020 ESR7 study centre) and University of Zagreb (Croatia, national study centre). Participating subjects signed the informed consent prior to data collection and were free to decline participation any time during the study. To ensure anonymity and data confidentiality, all data were collected and stored under specific codes. All methods were carried out in accordance with relevant project guidelines and regulations.

### Outcome measures and statistical analysis

The primary outcome was deprescribing potential of four commonly used medications in the community setting. To assess the deprescribing potential, deprescribing criteria for each medication were developed. Criteria were created based on available prescribing and deprescribing guidelines^22–24^. These included evidence-based explicit prescribing tools such as Beers^23^, LESS-CHRON^25^, START/STOPP criteria^26^, STOPPFrail^27^, and STOPPFall criteria^28^, PRISCUS 2.0 list^24^, as well as available medication-specific deprescribing guidelines^29–34^, clinical practice guidelines on treatment choices in older adults^35,36^, and summary of product characteristics^37^. Table 1 showcases deprescribing criteria for each of medication groups, while more detailed description of deprescribing criteria is available in Appendix file 1. The research team which participated in the EuroAgeism H2020 ESR 7 project, assessed the deprescribing potential. All researchers were familiarized with deprescribing criteria and their appropriate application. Junior researchers (community pharmacists with clinical background) collected and analysed the data. Senior researchers (clinical pharmacists with geriatric background) supervised the data analysis and application of criteria, and were available for discussion and final assessment of challenging cases. To ensure deprescribing potential was assessed in a standardized way at least two researchers needed to share agreement on selected criteria. For each patient, deprescribing potential was assessed by applying the deprescribing criteria while performing medication review and analysing the aforementioned collected data (pharmacist’s geriatric assessment). At least one deprescribing criterion had to be met for medication in question, for the patient to be considered a potential deprescribing candidate. Both potential clinically significant drug-drug interactions and adverse drug effects which could be associated with inappropriate use of certain medication were taken into account when considering safety concerns as deprescribing criteria. Detailed list of considered potential adverse drug effects can be found in Appendix 1. Reported symptoms, changes in cognitive status, falls, pain frequency and control, and diagnoses were assessed for analysis of potential adverse drug effects. For patients who reported *pro re nata* (PRN) use of medications; diagnoses, and the frequency and severity of symptoms (i.e. pain control, insomnia, reflux) was reviewed to determine the frequency of PRN use. Those reporting symptom frequency of less than couple of times a week were considered as true PRN users. Patients who did not know for how long they were using a certain medication (stating *“I do not know/remember”*(IDK)), were considered to be long-term users after diagnoses and symptoms review (i.e. reports symptoms of chronic pain but does not know when opioid/NSAID was started). Potentially clinically significant drug-drug interactions included analysis of interactions categorized by Lexicomp^®^ as D (therapy modification should be considered) and X (combination should be avoided) to avoid potential overestimation of the deprescribing potential due to safety concerns. Category C interactions, while clinically significant usually do not require dosage adjustments, and benefits of concomitant use usually overweigh the potential risks^38,39^, and therefor were not included in the analysis. If a patient was prescribed certain medication for other approved indications (i.e. diazepam/clonazepam for epilepsy, or muscle spasms) or for off-label indications, appropriateness for deprescribing was assessed based on diagnosis, safety criteria and frequency of use. In instances where data was missing, clinical assessment was unclear, or conflicting data was present, the application of specific deprescribing criteria was discussed. If feasible, a consensus was reached regarding the application of criteria, or if deemed impossible to assess, the case was identified as unassessable.

**Table 1:** Deprescribing criteria.

Criteria	PPI	NSAID	OPI	BZN
Lack of indication	appropriate indications: GERD, *H.pylori* eradication, ulcer disease, hypersecretory conditions, gastritis	Appropriate indications: chronic rheumatoid or short-term non-rheumatoid musculoskeletal pain	Resolution of pain/ definitive pain relieving intervention, lack of improvement in pain control	appropriate indications: insomnia disorders, anxiety disorders
	GI protection indicated, but no clear need/ low risk patient			
Inappropriately long use	> 4 weeks for symptomatic GERD > 8 weeks for reflux oesophagitis or peptic ulcer > 12 weeks for *H. pylori* ulcer disease	> 1 week for acute pain > 6 months for chronic pain^a^	> 6 months for non-cancer pain	> 4–8 weeks for insomnia disorders > 12 weeks for anxiety disorders
Inappropriate dose^b^	Use of higher than recommended gastroprotective dose	Use of higher than recommended daily dose	Use of more than 50 mg oMME for frail patients	Use of higher than recommended daily dose
	Prescribed for NSAID gastroprotection, but NSAID used PRN		Use of more than 90 mg oMME for non-frail patients	
Safety concerns*	Potentially clinically significant drug-drug interactions^c^	ADE associated with use	ADE associate with use	ADE associate with use
		Risk factors which could be exacerbated by NSAID use		Potentially clinically significant drug-drug interactions
		Potentially clinically significant drug-drug interactions^c^	Potentially clinically significant drug-drug interactions^c^	Frail patients

PPI—proton pump inhibitors, NSAID—nonsteroidal anti-inflammatory drugs, OPI—opioid analgesics, BZN—benzodiazepine receptor agonists, GI—gastrointestinal, GERD—gastroesophageal reflux disease, PRN—pro re nata use, ADE—adverse drug effects, oMME—oral morphine milligrams equivalent, ^b^inappropriate dose of each medication can be found in Appendix file 1 (inappropriate dose included inappropriate dosing regimen such as dosing too frequently), ^c^potential clinically significant drug-drug interactions identified as D or X as assessed by Lexicomp^®^, ^a^more than 6 months for patients prescribed adequate gastroprotection, *Included contraindications for use.

Descriptive statistics were used to analyse sociodemographic data, and a chi-squared test was used to analyse differences in frequencies between groups. To explore the effects of age, gender, number of medicines, number of diagnoses, self-reported health, frailty score, and healthcare utilization on the likelihood of deprescribing potential a binary logistic regression was performed. For the purposes of the logistic regression, nominal variables self-reported health, healthcare utilization, and frailty score were dichotomised. Categories ”very poor” and ”poor” formed “poor”, and “moderate”, “good”, and “very good” formed “good” for the variable self-reported health. Variable frailty score was dichotomised into “frail” (frailty score from 4 to 9) and “non frail” (frailty score from 1 to 3), while healthcare utilisation (combined variable of hospitalisations and emergency department visits) was dichotomised into “utilisation within the last 12 months” and “no utilisation in the last 12 months”. For all analyses, a value of *p* < 0.05 was considered statistically significant. All analyses were performed using IBM SPSS Statistics for Windows, Version 26.0. (IBM Corp., Armonk, NY, USA).

### Ethics approval and consent to participate

Ethical approval for this study was obtained from the Ethical committees of the Charles University (Czech Republic, EuroAgeism H2020 ESR7 study centre) and University of Zagreb (Croatia, national study centre). Participating subjects signed the informed consent prior to data collection, and were free to decline participation any time during the study. To ensure anonymity and data confidentiality, all data were collected and stored under specific codes. All methods were carried out in accordance with relevant project guidelines and regulations.

## Results

### Participants characteristics

In total 388 older adults participated, of which 269 (69.3%) used at least one of the medications of interest. Almost one third of all participants used a proton pump inhibitor (n = 122, 31.4%), and almost 40% used a BZN (n = 154, 39.7%). Use of NSAID and opioid analgesics was noted in 111 (28.6%) and 60 (15.5%) participants, respectively. Most commonly used medication combinations were a PPI and a BZN (n = 32, 8.2%), PPI, NSAID and BZN (n = 24, 6.2%), and a combination of a NSAID and a BZN (n = 23, 5.9%). Only three participants used all four types of medications simultaneously. Additional information on participants’ characteristics can be found in Table 2.

**Table 2:** Participants’ characteristics.

Characteristic	N = 388 participants
Age (median, IQR)	73 years (IQR 69–79.75)
Gender (women, n; %)	247; 63.7%
Region (n; %)	
North–west continental	144; 37.1%
North–east continental	125; 32.2%
Coastal	119; 30.7%
Number of medicines (median, IQR)	6 (IQR 4–8)
Number of diagnosis (median, IQR)	5 (IQR 3–8)
Last hospitalization (n; % of participants)^a^	
Within the last 12 months	51; 13.9%
More than 12 months ago	317; 86.1%
Emergency department visits (n; % of participants)^a, b^	
Yes	97; 25.1%
No	290; 74.9%
Utilization of other healthcare services (n; % of participants)^a, b^	
Yes	54; 14.1%
No	328; 85.9%
Self-reported health status (n; % of participants)^a^	
Very poor	6; 1.6%
Poor	36; 9.4%
Moderate	151; 39.0%
Good	142; 36.7%
Very good	52; 13.4%
Frailty score (n; % of participants)	
Non frail (score 3 or less)	285; 74.2%
Frail (score 4 or higher)	99; 25.8%
Length of medicine use (median, IQR)^c^	
PPI	4 years (IQR 2–6)
NSAID	3 years (IQR 2–5 years)
OPIOID	2.5 years (IQR 2–5 years)
BZN	5 years (IQR 2–10 years)

IQR—interquartile range, ^a^calculated from non-missing values (missing values less than 5%), ^b^within the previous 12 months (other healthcare services include services such as physiotherapy, palliative care, rehabilitations, home care…), ^c^patients stating IDK for length of medication use: 41 for PPI, 52 for NSAID, 22 for opioids, and 61 for BZN.

### Potential for deprescribing

Based on deprescribing criteria more than half of patients (n = 216, 55.7%) would be candidates for deprescribing, with 33.5% for one medicine, 18.8% for two medicines, and 3.4% for three medicines. When it comes to specific type of medicine, 31.1% of PPI users, 74.8% of NSAID users, 75% of opioid users, and 96.1% of BZN users would be candidates for deprescribing. Information on criteria which participants satisfied for deprescribing of particular medicine can be found in Table 3 and more detailed descriptive statistics is available in Appendix file 2. In 52.6% (n = 55) of BZN users, 30% (n = 18) OPI users, and 6.3% (n = 7) NSAID users adverse effects could be associated with use of other medicines. Half of PPI users reported gastrointestinal symptoms regardless of PPI use, and 17.2% (n = 21) should use a PPI for gastroprotection but had it prescribed for another diagnosis.

**Table 3:** Analysis of deprescribing criteria for each therapeutic class.

Criteria^a^		PPI	NSAID	OPI	BZN
Total number of deprescribing candidates (n, % of users)		n = 38/122 (31.1%)	n = 83/111 (74.8%)	n = 45/60 75.0% (75.0%)	96.1% n = 148/154 (96.1%)
Lack of indication (n, % of users)		n = 9/122 (7.4%)	0	18.3% n = 11/60 (18.3%)	n = 38/154 (24.7%)
Inappropriately long use (n, % of users)		n = 32/122 (26.2%)	n = 58/111 (52.3%)	n = 42/60 70.00% (70.00%)	n = 94/154 (61.0%) for insomnia use n = 73/154 (47.71%) for anxiety use
Inappropriate dose (n, % of users)		n = 20/122 (16.4%) inappropriately high gastroprotective dose	n = 19/111 (17.1%) higher than recommended daily dose	0	n = 26/154 (17.0%) higher than recommended daily dose
Safety concerns (n, % of users)	Potential clinically significant DDI	n = 3/122 (2.5%)	n = 36/111 (32.4%)	n = 31/60 (51.7%)	n = 39/154 (25.5%)
	Presence of ADE		n = 45/111 (40.5%)	n = 32/60 (56.3%)	n = 81/154 (52.6%)
	Pther safety concerns		n = 35/111 (30.97%) with factors which could be exacerbated by NSAID use	0	n = 56/154 (36.6%) frailty score 4 and above

^a^Patient could meet multiple deprescribing criteria for a single therapeutic class, PPI—proton pump inhibitors, NSAID—nonsteroidal anti-inflammatory drugs, OPI-opioid analgesics, BZN—benzodiazepine receptor agonists, DDI—drug-drug interaction, ADE—adverse drug effects, pro re nata use was noted in 4.09% (n = 5/122) PPI users, 18.92% (n = 21/111) NSAID users, 23.33% (n = 14/60) OPI users, and in 26.80% (n = 41/154) BZN users.

Siginificant values are in bold.

In a univariate analysis, several factors were found to be associated with a higher potential for deprescribing, namely female gender, six or more diagnoses, and poor self-reported health status. Women (71.3%) were more likely to need to have medicines deprescribed than men (28.7%)(χ^2^ (1) = 12.283, *p* < 0.001), and those with six or more diagnosis (58.9%) were more likely to need to have medicines deprescribed than those with five or less (46.2%) (χ^2^ (1) = 7.088, *p* = 0.008). Those who reported being of poor health (88.1%) were more likely to need to have medicines deprescribed than those who reported being of good health (51.9%)(χ2 (1) = 19.907, *p* < 0.001). Healthcare utilization was more prevalent in those needing to have medication deprescribed, with those who needed to have one or more medicines deprescribed being more likely to experience emergency department visit in the previous 12 months (31.2% vs.17.4%; χ^2^ (1) = 9.578, *p* = 0.002) or experience a hospitalisation (χ^2^ (4) = 12.206, *p* = 0.016). No statistically significant difference was found in deprescribing potential between different age groups or regions.

### Predictors of potential for deprescribing

A binary logistic regression model was employed to examine potential predictors for an increased deprescribing potential. The model included several variables: age, number of medicines, number of diagnoses, gender, healthcare utilization, frailty score, and self-reported health. Among these variables, gender, number of medicines and self-reported health emerged as statistically significant predictors of deprescribing potential. Women had 2.58 times higher odds (aOR = 2.58; 95% CI = 1.59–4.18) of requiring deprescribed than men. The odds ratio for the number of medicines (aOR = 1.29; 95% CI = 1.17–1.44) indicated that the higher the number of medicines taken by a patient, the higher the likelihood of needing deprescribing. Participants who reported poor health had 5.14 times higher odds (aOR = 5.14; 95% CI = 1.73–15.25) of needing deprescribing compared to those who reported good health (Table 4).

**Table 4:** Deprescribing potential binary logistic regression analysis.

Independent variable	aOR	95% CI		*p* value
Age	1.00	0.98	1.00	0.813
Number of medicines	**1.29**	**1.17**	**1.44**	** < 0.001**
Number of diagnoses	0.93	0.84	1.02	0.128
Women	**2.58**	**1.59**	**4.18**	** < 0.001**
Utilization of healthcare in the previous 12 months^a^	1.30	0.78	2.16	0.322
Frail patients^b^	1.22	0.67	2.22	0.506
Poor self-reported health	**5.14**	**1.73**	**15.25**	** < 0.001**

The logistic regression model was significant (*p* < 0.001) with a good model fit (Hosmer–Lemeshow test χ^2^ (8) = 3.037 *p* = 0.932). The model explained 24.20% of the variance in deprescribing potential and correctly predicted 68.0% of cases.

^a^dichotomized variable with categories: utilization in the previous 12 months and utilization more than 12 months ago, ^b^dichotomized variable with categories: score 1–3 indicating non frail patients and score 4–9 indication frail patients, aOR—adjusted odds ratio, CI—confidence interval.

Significant values are in bold.

## Discussion

More than half of all participants were candidates for deprescribing, and the most common criteria for deprescribing were inappropriately long use followed by safety concerns, and lack of indication. Similar patterns were found for pharmacists’ deprescribing recommendations in the tertiary hospital in Singapore and long term care facilities settings in Australia^40,41^, highlighting consistent inappropriate prescribing patters in older adults’ pharmacotherapy regardless of setting and geographical location.

The lowest number of deprescribing candidates were PPI users due to high number of patients reporting symptoms regardless of PPI use. Even though older adults often require pharmacotherapy with PPIs, evidence suggests low-dose, or on-demand use can be a reliable strategy to reduce the rate of unnecessary high-dose or prolonged PPI therapy while providing adequate symptom control^42,43^. Although reducing existing or potential harm is one of the main goals of deprescribing, when it comes to analgesics, it is important to maintain pain control even after medication withdrawal. Large number of NSAID users were candidates for deprescribing due to safety concerns, and opioid users were confronted with a twofold setback, of inappropriately long use and safety concerns. Pharmacist-led deprescribing interventions can lead to a decrease in use of NSAIDs and still effectively manage pain^44,45^. Opioids can be efficient in improving pain in the short-term, but long-term therapy may actually worsen the impact of chronic pain on quality of life due to low efficacy and adverse effects^46^. Multidisciplinary care programmes seem to be effective in opioid deprescribing^47^, but additional evidence is needed to assess the most suitable type of intervention. An overwhelming number of BZN users are candidates for deprescribing, mostly due to inappropriately long use and adverse effects. Deprescribing BZN can be challenging for both patients and healthcare providers, but when provided with a non-pharmacological support can be successful^48,49^. For each medication group analysed in this study, there are substantial evidence and guidelines at healthcare providers’ disposal, which should be tailored to individual patient’s needs and utilized during patient care.

Several factors were identified as potential predictors for increased need to have medicines deprescribed, including female gender, reporting poor health, and using multiple medications. Besides keeping in mind pharmacodynamic and pharmacokinetic differences between men and women, healthcare providers should consider other factors which could influence adequate provision of healthcare to men and women. A review by Rochon et al. explores the importance of sex and gender differences in providing care when it comes to polypharmacy and potential deprescribing, highlighting how women are more likely reach to old age, be exposed to inappropriate prescribing and polypharmacy, and be at risk or drug-related adverse events^50^. Women are also more likely to consider the impact of medication when it comes to the decision to agree with deprescribing, while men find the impact of physician more important^51^. Self-reported health, which is negatively associated with polypharmacy^52^, can be used in predicting short-term mortality risk among older adults^53^, and has been identified as one of the priority outcomes in deprescribing research^54,55^. There is lack of evidence on the effect of deprescribing on self-reported health, but results of one study suggest that deprescribing can have a positive effect on increasing and/or sustaining levels of self-reported health^56^. Deprescribing can have a positive impact on other clinical outcomes which can then affect self-perception of health, such as mental health status, function, or frailty^57^. Furthermore, higher the use of medications, higher the odds participant will need medications deprescribed. When examining the deprescribing potential of four medication groups, more than one fifth of participants were suitable candidates for deprescribing multiple medications. Polypharmacy has been recognised as a risk factor for negative outcomes, and where appropriate polydeprescribing (the simultaneous deprescribing of multiple medications) could be recommended to quicken the process without compromising patient safety^58^. For healthcare providers polydeprescribing enables tackling multiple medications at once as deprescribing priorities, which can potentially lead to earlier improvement in outcomes for those eligible patients who are comfortable with accepting discontinuation of multiple medications. Healthcare providers should carefully consider patients who exhibit multiple factors associated with increased deprescribing potential.

As deprescribing is a patient-centred process and requires shared decision-making, it is important to evaluate patients’ opinions and attitudes before suggesting deprescribing. Evidence suggests patients are willing to have medicines deprescribed^59,60^, but actual number of patients who accept deprescribing could be lower^61^. No difference was found between different age groups regarding deprescribing potential in this study, and every eligible patient should be offered deprescribing. Nevertheless, there is a potential difference in acceptance of deprescribing suggestions among different age groups, with very old adults expressing satisfaction with pharmacotherapy and not seeing any need for medication withdrawal^62,63^, which should be taken into account when providing care for older adults.

Several limitations need to be stated. Analysis of safety concerns, namely the effect of found potentially clinically significant interactions needs to be interpreted with caution, as interactions should be assessed and confirmed at point-of-care and include detailed clinical interpretation with extensive clinical data, which could not have been collected in its entirety with the used questionnaire. For those reasons, the research team focused on interactions which could be interpreted based on collected data and patient context. Another limitation in this study is the lack of a shared medical electronic records across different healthcare levels, and lack of electronic medical record available in the community pharmacy. As a result, the accuracy of the data used for analysis relied on information collected directly from the patient and the medical documentation provided by the participant to the researchers. Potential for deprescribing was assessed for four medication groups, which could be viewed as a limitation, as true need for deprescribing is underestimated. Whereas it would have been interesting to explore the deprescribing potential of other commonly used medications, such as antihypertensives, antidepressants, antipsychotics, or other fall risk increasing medications, the data did not present enough clinical information to adequately assess disease control and subsequent deprescribing potential. Nevertheless, these four medication groups represent most commonly used medicines in the sample’s population^64^, and were most commonly recognised as inappropriate medications needing deprescribing^2,65^. On the other hand, use of pharmacist’s geriatric assessment as well as medication review with detailed deprescribing criteria ensured deprescribing potential was judged considering all important aspects of patients’ health. Results of pharmacist’s geriatric assessment with the analysis of deprescribing potential should be a part of a more encompassing interdisciplinary approach, involving general practitioners and specialists such as geriatricians, in order to verify and position the findings in a clinical context of the patient in question to reach the desired therapeutic goal. Comprehensive geriatric assessment has been proven to be a useful method for identifying deprescribing targets, and a combination of clinical geriatric assessment and collaborative medication review can result in positive effects on health-related quality of life^66,67^.

Additional limitations include analysis performed on data collected for one participating country from the EuroAgeism H2020 project, and cross-sectional study design for which causal relationships cannot be confirmed. However, this sample adequately represents patients from this high-income Central and Eastern European country, with relatively high use of potentially inappropriate medications among older adults in the community setting^68^ and average frailty prevalence^69^. The lack of data on deprescribing potential in community-dwelling older adults in Central and Eastern Europe, including other participating countries, further emphasizes the importance of the results obtained from this study. While this study explores the potential for deprescribing in the community-dwelling adults, it can be assumed the need for deprescribing is even more pronounced in secondary or tertiary settings, and in long-term care facilities. Deprescribing potential assessed using available deprescribing guidelines in a retrospective study on hospitalized older patients showed almost three quarters of patients were deprescribing candidates^70^. There is a need for additional research and comparative studies (within Europe and worldwide) to get a better insight: into the deprescribing potential among vulnerable patient groups, as well as to assess the availability of medication management services, particularly in healthcare settings unfamiliar with deprescribing. This can help identify differences and variations in prescribing practices, as well as highlight the opportunities and challenges for implementing deprescribing into everyday practice. Results of this study additionally highlight the importance of community pharmacists’ involvement in providing safe and personalized multidisciplinary geriatric care and underscore the possibilities of implementing a more active role of community pharmacists in achieving better outcomes for older adults.

Further research is necessary to establish how identified factors influence provision and success of a deprescribing intervention, especially when it comes to clinical and patient-related outcomes, such as self-reported health status. Potential target subpopulation could be women who are exposed to inappropriate polypharmacy and are expressing poor self-reported health.

## Conclusion

A significant proportion of older adults are eligible candidates for deprescribing one or more medicines, with a particular emphasis on the deprescribing potential of benzodiazepines. followed by analgesics. Polypharmacy and poor self-reported health, as well as being a woman, have been identified as factors contributing to increased deprescribing potential. Timely action towards reducing the use of commonly prescribed potentially inappropriate medications is needed to increase patient safety and contribute to healthy ageing. Personalised approach can be achieved through pharmacist’s geriatric assessment and deprescribing-focused medication review.

### Supplementary Information

Supplementary Information.

## Data Availability

The datasets used and/or analysed during the current study are available from the corresponding author upon reasonable request.
