# Author Correction: Deprescribing potential of commonly used medications among community-dwelling older adults: insights from a pharmacist’s geriatric assessment

**DOI:** 10.1038/s41598-024-60073-y

**Published:** 2024-04-22

**Authors:** Iva Bužančić, Margita Držaić, Ingrid Kummer, Maja Ortner Hadžiabdić, Jovana Brkić, Daniela Fialová

**Affiliations:** 1 City Pharmacies Zagreb, Kralja Držislava 6, Zagreb, Croatia; 2 Faculty of Pharmacy and Biochemistry, Center for Applied Pharmacy, University of Zagreb, Ante Kovačića 1, 10 000 Zagreb, Croatia; 3 Department of Social and Clinical Pharmacy, Faculty of Pharmacy in Hradec Králové, Charles University, Akademika Heyrovského 1203/8, Hradec Králové, Prague, Czech Republic; 4 Department of Social Pharmacy and Pharmaceutical Legislation, Faculty of Pharmacy, University of Belgrade, 450 Vojvode Stepe Street, Belgrade, Serbia; 5 Department of Geriatrics and Gerontology, 1st Faculty of Medicine in Prague, Charles University, Kateřinská 32, Prague, Czech Republic

Correction to: *Scientific Reports* 10.1038/s41598-024-56780-1, published online 14 March 2024

The original version of this Article contained an error in the Funding section.

“All the research work was funded by the EuroAgeism H2020 project (ESR7 project), supported by the European Union research and innovation program under the Grant Agreement of the Marie Skłodowska-Curie Foundation Number MSCF-ITN-764632. Research works of Assoc. Prof. Daniela Fialová, PharmD, Ph.D. and members of her research team were supported by the Grants: InoMed, Reg. No CZ.02.1.01/0.0/0.0/18_069/0010046, the European Horizon 2020 I-CARE4OLD Grant No 965341, START/MED/093 EN.02.2.69/0.0/0.0/19_073/0016935, SVV 260 551 Grant and Cooperatio research program of the Faculty of Pharmacy, Charles University (Research Unit: “Ageing, Polypharmacotherapy and Changes in Therapeutic Value of Drugs in the Aged’’, KSKF-I.), and NETPHARM project CZ.02.01.01/00/22_008/0004607. The funding organizations had no role in the design and conduct of the study; collection, management, analysis, and interpretation of the data; preparation, review, or approval of the manuscript; and decision to submit the manuscript for publication.”

now reads:

“Publication of this work was supported by the I-CARE4OLD project that has received funding from the European Union’s Horizon 2020 research and innovation programme under the grant agreement No. 96534. Views and opinions expressed are however those of the authors only and do not necessarily reflect those of the European Union. Neither the European Union nor the granting authority can be held responsible for them. More information on the I-CARE4OLD project can be found at http://www.icare4old.eu and https://cordis.europa.eu/project/id/965341. Except secondary analyses and works on the publication, data collection and teamwork were funded also by the EuroAgeism H2020 project (ESR7 project), supported by the European Union research and innovation program under the Grant Agreement of the Marie Skłodowska-Curie Foundation Number MSCF-ITN-764632. Research works of Assoc. Prof. Daniela Fialová, PharmD, Ph.D. and members of her research team were supported by grants: START/MED/093 EN.02.2.69/0.0/0.0/19_073/0016935, SVV 260 551, Cooperatio research program of the Faculty of Pharmacy, Charles University (Research Unit: “Ageing, Polypharmacotherapy and Changes in Terapeutic Value of Drugs in the Aged’’, KSKF-I.), and NETPHARM project CZ.02.01.01/00/22_008/0004607.”

The original Article has been corrected.

