# Supplementary material for: Deprescribing potential of commonly used medications among community-dwelling older adults: insights from a pharmacist’s geriatric assessment
Source: Sci Rep. 2024 Mar 14;14:6235. doi: 10.1038/s41598-024-56780-1 (PMC10940601; doi:10.1038/s41598-024-56780-1)
Supplement: Supplementary file 1 — Supplementary Information. [file 41598_2024_56780_MOESM1_ESM.docx]

Deprescribing Potential of Commonly Used Medications among community-dwelling Older Adults: Insights from a Pharmacist’s Geriatric Assessment

**Authors**: Iva Bužančić, (ORCID ID: 0000-0002-4140-8657) ^1,2,^*; Margita Držaić, (ORCID ID: 0000-0002-1539-4482) ^1,2,^*; Ingrid Kummer, (ORCID ID: 0000-0003-0205-1576) ^3^; Maja Ortner Hadžiabdić, (ORCID ID: 0000-0003-1578-9764) ^2^ ; Jovana Brkić, ^3,4^; Daniela Fialová, (ORCID ID: 0000-0001-5638-9690) ^3,5^

^1^ City Pharmacies Zagreb, Kralja Držislava 6, Zagreb, Croatia

^2^ University of Zagreb, Faculty of Pharmacy and Biochemistry, A. Kovačića 1, Zagreb, Croatia

^3^ Department of Social and Clinical Pharmacy, Faculty of Pharmacy in Hradec Králové, Akademika Heyrovského 1203/8, Hradec Králové, Charles University, Prague, Czech Republic

^4^ Department of Social Pharmacy and Pharmaceutical Legislation, Faculty of Pharmacy, University of Belgrade, 450 Vojvode Stepe Street, Belgrade, Serbia

^5^ Department of Geriatrics and Gerontology, 1st Faculty of Medicine in Prague, Charles University, Kateřinská 32, Prague, Czech Republic

*shared first authorship; these authors contributed equally

email addresses: Iva Bužančić- buzanciciva@gmail.com

Margita Držaić- margitadrzaic@gmail.com

Ingrid Kummer- ingrid.kummer2@gmail.com

Maja Ortner Hadžiabdić- mortner@pharma.hr

Jovana Brkić- jovanabrkic37@gmail.com

Daniela Fialová- fialovad@faf.cuni.cz

**Corresponding author**: Assoc. Prof. Maja Ortner Hadžiabdić, MPharm, PhD

Center for Applied Pharmacy

University of Zagreb

Faculty of Pharmacy and Biochemistry

Ante Kovačića 1, 10 000 Zagreb, Croatia

E-mail: mortner@pharma.hr

Deprescribing Potential of Commonly Used Medications among Community-Dwelling Older Adults: Insights from a Pharmacist-led Geriatric Assessment-APPENDIX FILE 1

Proton Pump Inhibitors (PPI) Deprescribing criteria^1,2^:

PPI available: pantoprazole, omeprazole, esomeprazole, rabeprazole, lansoprazole

Deprescribing can include stopping, stepping down, or reducing doses. Stopping can be done either via abrupt discontinuation or a tapering regimen. Stepping down involves abrupt discontinuation or tapering of the PPI followed by prescription of an H2 RA (any H2 RA at any approved dose and dosing interval according to the drug monograph)

Reducing includes the following subcategories:

-Intermittent PPI use, which is defined by the Canadian Consensus Conference as “daily intake of a medication for a predetermined, finite period (usually two to eight weeks) to produce resolution of reflux-related symptoms or healing of oesophageal lesions following relapse of the individual’s condition”

-On-demand PPI use, which is defined by the Canadian Consensus Conference as “the daily intake of a medication for a period sufficient to achieve resolution of the individual’s reflux-related symptoms; following symptom resolution, the medication is discontinued until the individual’s symptoms recur, at which point, medication is again taken daily until the symptoms resolve”

-Low dose, which is a reduction from a standard dose to a maintenance dose

For adults (>18 y) with upper gastrointestinal (GI) symptoms, who have completed a minimum 4-week course of PPI treatment, resulting in resolution of upper GI symptoms, it is recommended to decrease the daily dose or stop and change to on-demand (as needed) use. Alternatively, it can be suggested to consider an H2 RA as an alternative to PPIs.

Risk factors for GI bleed in antiplatelet user:

- concomitant use of another antiplatelet
- previous peptic ulcer disease
- concomitant therapy with nonsteroidal anti-inflammatory drug (NSAID)
- H.pylori infection
- >70 years of age

Risk factors for GI bleed in vitamin K antagonist (VKA) and direct oral anticoagulant (DOAC) user:

- >65 years of age (VKA) or >75 years of age (DOAC; >70 years of age for dabigatran)
- previous GI bleed or ulcer disease
- liver cirrhosis (VKA) or kidney failure (DOAC)
- diverticulosis of the colon (VKA)
- concomitant use of lipid-lowering agents (VKA)
- concomitant use of NSAIDs, acetylsalicylic acid (ASA), or COX-2
- concomitant use of an antiplatelet

General factors for GI bleed:

| **high risk factors for GI bleed (one or more)** | **moderate risk factors for GI bleed (two or more)** |
| --- | --- |
| history of GI ulcer or ulcer complication | dyspepsia or GERD symptoms |
| previous upper gastrointestinal or lower gastrointestinal bleeding | concomitant use of medicines known to increase risk of GI bleeds (oral corticosteroids) |
| dual antiplatelet therapy | heavy smoking (more than 10 cigarettes a day) |
| concomitant use of anticoagulant/antiplatelet with:   - orally administered corticosteroids - with ASA or other antiplatelets (including low dose ASA) - with high dose of NSAID or multiple NSAID therapy (i.e. >2400mg/ibuprofen) | excess alcohol consumption (more than 8 drinks per week for women, more than 15 drinks per week for men) |
| patients >70 years of age receiving antiplatelet treatment (including low dose ASA) | patients >65 years |

1. **Lack of indication for use PPI** **and patient does not report any symptoms** (such as eGERD, h.pylori eradication, ulcer disease, Zollinger-Ellison syndrome or other hypersecretory conditions, short-term treatment of acid reflux conditions or gastritis)
2. **Inappropriately long use and patient does not report any symptoms** (indication for use is present, but the length of use is inappropriate: for reflux oesophagitis longer than 8 weeks, for symptomatic GERD longer than 4 weeks, for h.pylori ulcer disease for longer than 12 weeks, for peptic ulcer treatment longer than 8 weeks) without appropriate indication for long-term use (Barret’s oesophagus, chronic NSAID use with increased bleeding risk, sever oesophagitis, or bleeding GI ulcer)
3. **GI protection indicated**^3^, **but patient presented without clear need for such treatment**/ **patient has low risk**^4,5^.
   1. In case of chronic non-COX-selective NSAID use, PPI gastroprotection is indicated for patients with moderate (2 risk factors) or high risk of GI bleeding (history of GI ulcer, or 3 and more risk factors).
   2. In case of chronic selective COX-2 inhibitor use, PPI gastroprotection is indicated in high risk patients
   3. In case of anticoagulant and antiplatelet medications use, PPI gastroprotection is indicated in patients with moderate (2 risk factors) or high risk of GI bleeding (history of GI ulcer, or 3 and more risk factors)
   4. In case of use of orally administered corticosteroids, PPI gastroprotection is recommended for patients with history of GI ulcer, and/or concomitant use of NSAIDs
4. **Prescribed gastroprotective dose of PPI is too high**
   1. Recommended doses are: 10mg/day for rabeprazole, 20mg/ day for esomeprazole, pantoprazole, and omeprazole, and 30mg/day for lansoprazole
5. **PPI prescribed for NSAID gastroprotection**, **but NSAID used as need**
6. **Safety concerns**
   1. potential clinically significant drug-drug interactions (D or X as assessed by Lexicomp®)

Non-Steroidal Anti-Inflammatory Drugs (NSAID) deprescribing criteria^6–11^

NSAIDs available: acemetacin, ibuprofen, ketoprofen, diclofenac, dexketoprofen, indomethacin, naproxen, celecoxib, etoricoxib, piroxicam, meloxicam

Generally, there is no tapering required when deprescribing NSAIDs for osteoarthritis. Where there is an underlying inflammatory condition (e.g. rheumatoid arthritis) there may be an increase in pain after cessation. This may be attenuated by slow dose reduction.^6^

1. **Lack of indication for chronic use NSAIDs**^6,8,9^ (approved indications include chronic rheumatoid and non-rheumatoid musculo-skeletal pain such as arthritis, osteoarthritis, tendinitis)
   1. chronic use (excluding *pro re nata* use) following NSAID prescription for acute pain indications (i.e. toothache, headache, migraine….)
2. **Safety concerns** ^7,8^(regarding chronic use)
   1. patient presents with adverse effects which could be associated with NSAID use (oedema, dizziness, acid reflux/dyspepsia/epigastric pain/heartburn, nausea and vomiting, abdominal pain, diarrhoea, rash, vertigo, hypertension, heart palpitations)
   2. patient has at least 1 risk factor which could be exacerbated by NSAID use (diagnosed within past 12 months)- past, active or suspected GI bleed/ulcer, heart failure (NYHA stage II-IV), ischaemic heart disease (diclofenac, celecoxib, etoricoxib), atherosclerotic cardiovascular disease (diclofenac, celecoxib, etoricoxib), renal disease (GFR< 30ml/min; <60ml/min for dexketoprofen), liver failure, inflammatory bowel disease (ulcerative colitis or Crohn disease), coagulation diseases, history of ASA/NSAID exacerbated asthma/allergies/rhinitis/nasal polyposis
   3. potential clinically significant drug-drug interactions (D or X as assessed by Lexicomp®, such as combinations with warfarin, NOACs, antiplatelet, orally administrated corticosteroids)
3. **Appropriate indication but inappropriate dose** (deprescribing through dose reduction or *pro re nata* use (PRN)). Guidelines for the management of osteoarthritis of the hand, hip, and knee recommended considering topical NSAIDs prior to use of oral NSAIDs (strongest recommendations for treatment of knee pain) as well as using as low as possible oral doses of NSAIDs.^9^ For certain NSAIDs research shows comparable efficacy for the lower dosages in comparison to higher. Summaries of product characteristics recommend lowering the dose 25-50% and/or using the lowest effective dose for the shortest duration possible when treating older adults: ibuprofen (1200mg vs 2400mg), naproxen (750mg vs 1000mg), diclofenac (75mg vs 150mg), dexketoprofen (50mg vs 75mg), ketoprofen (100mg vs 200mg), acemetacin (120mg vs 180mg), indomethacin (150mg vs 200mg), meloxicam (7.5mg vs 15mg), piroxicam (10mg vs 20mg), etoricoxib (60mg vs 90mg) and celecoxib (200mg vs 400mg).
   1. patient with moderate risk factors including concomitant therapy with diuretics, ACE- inhibitors, alcohol over use, smoking, GERD, h.pylori infection^6^
4. **Inappropriately long use of NSAIDs**^10,12^**.** Based on PRISCUS 2.0 list, Beers’ criteria and START/STOP criteria inappropriate use of NSAID was considered as
   1. >1 week for acute pain
   2. >6 months for chronic pain in patients with adequate gastroprotection
   3. >3 months for chronic pain in patients without adequate gastroprotection

Opioid analgesics Deprescribing criteria^13–17^

Opioid analgesic available: tramadol, codeine, oxycodone ± naloxone, tapentadol, fentanyl, morphine, buprenorphine

Long-term opioid analgesic use (> 6 months) for non-cancer pain is not recommended in older adults.

Precautions should be implemented when dosage ≥50 mg oral morphine milligrams equivalents (oMME), doses above 90 mg oMME should be avoided. [approximate dose for concern: 250mg/day tramadol, 125mg/day tapentadol, 30mg/day oxycodone, 25mcg/h fentanyl transdermal]

LONG TERM USE WITHOUT CLEAR BENEFIT: tapering by 5-25% of daily dose each month (tapering through 3-9 months)

END OF AGREED TRIAL or FAILURE OF TREATMENT: tapering by 5-25% of daily dose each week

SIGNIFICANT ADVERSE EFFECTS: immediate cessation and pharmacological treatment of withdrawal symptoms or daily step-wise reduction 5-25% every day or every week

1. **Lack of indication for opioid use**^18^
   1. resolution of pain or definitive pain relieving intervention (i.e. joint replacement)
   2. lack of improvement in pain control (less than 30% reduction in pain) (patients reporting moderate, severe or very severe pain regardless of opioid use) in 4 weeks
2. **Inappropriately long use of opioids**
   1. use >6 months for non-cancer pain is not recommended in older adults
3. **Safety concerns** ^19^
   1. patient presents with adverse effects which could be associated with opioid use (falls, dizziness, drowsiness, orthostatic hypotension, dry mouth, itchiness, nausea, urinary retention, constipation, respiratory depression, cognitive impairment, mood changes)
   2. potential clinically significant drug-drug interactions (D or X as assessed by Lexicomp®, such as CNS depressants) which could increase risk of negative outcomes
4. **Inappropriate dose or dosing**
   1. more than 50mg oMME for frail elderly (those with clinical frailty score of 4 and above)
   2. more than 90 mg oMME for non-frail elderly

Benzodiazepine receptor agonists Deprescribing criteria^20–27^

BZN available: alprazolam, diazepam, lorazepam, oxazepam, nitrazepam, bromazepam, clonazepam, midazolam, zolpidem, zopicolne

Consider deprescribing if prescribed for primary insomnia or secondary insomnia where the primary underlying comorbidity is managed. Those older than 65 years should avoid benzodiazepine as first line therapy. For those 18 to 64 years deprescribing should be limited to 4 weeks of therapy.

Benzodiazepines can be continued in those using them for other indications such as unmanaged anxiety or depression, physical or mental condition that may be causing or aggravating insomnia, during alcohol withdrawal, for other sleeping disorders such as restless leg syndrome, or if benzodiazepines are effective specifically for anxiety. Underlying conditions should be treated, and use of substances worsening insomnia should be limited and minimized.

Taper slowly in collaboration with patient, for example 15-25% every 1-4 weeks, and if possible, 12.5% reductions near end and/or planned drug-free days (plan 6-8 weeks of tapering). One option (for patients using benzodiazepines for insomnia) is to advise not taking the agent one night a week for a week (or two), two nights the next week or two, three nights in the next, etc. Monitor patient every 1-2 weeks for duration of tapering. If dosage forms do not allow 25% reduction, consider 50% reduction initially and using drug-free days during latter part of tapering. If patients develop significant intolerant withdrawal or discontinuation symptoms, a return to the previous tapering step for a longer period of time (e.g. a month) often allows for a reattempt of dose reduction.

1. **Lack of indication for use of benzodiazepines** (insomnia disorders, anxiety disorder managed with other medications)
   1. patient using two or more different benzodiazepines at the same time
2. **Inappropriately long use of benzodiazepines**
   1. use longer than 4-8 weeks for insomnia disorders (including time for tapering)
   2. use longer than 12 weeks for anxiety disorders (including time for tapering)
3. **Safety concerns**^28^
   1. patient presents with adverse effects which could be associated with inappropriate benzodiazepine use (falls, dizziness, drowsiness, hypotension, blurred vision, confusion, cognitive impairment, mood changes, ataxia, light-headedness, headaches, respiratory suppression, oversedation, nightmares, impaired concentration)
   2. frail patients (those with clinical frailty score of 4 and above)
   3. potential clinically significant drug-drug interactions (D or X as assessed by Lexicomp®, such as CNS depressants) which could increase risk of negative outcomes
4. **Inappropriate dose**^20,26^
   1. patient using a higher than recommended daily dose for older adults (dose expressed as mg/day and based on information available from national summary of product characteristics and Lexicomp database)

|  | **alprazolam** | **diazepam** | **bromazepam** | **clonazepam** | **lorazepam** | **midazolam** | **nitrazepam** | **oxazepam** | **zolpidem** | **zopiclone** |
| --- | --- | --- | --- | --- | --- | --- | --- | --- | --- | --- |
| **insomnia**  **mg/day** | ***NA*** | **5** | **3** | ***NA*** | **0.5-1** | **7.5** | **5** | **20** | **5-10** | **3.75-7.5** |
| **anxiety**  **mg/day** | **0.75** | **15** | **3** | **0.5** | **0.5-1** | ***NA*** | ***NA*** | **10** | ***NA*** | ***NA*** |

ADDITIONAL CONSIDERATIONS^29,30^:

Under lack of indication all stated contraindications for use of particular medication need to be taken under consideration (*i.e.* heart failure and chronic NSAID use, respiratory suppression and opioids).

If a patient was prescribed certain medication for other approved indications (*i.e.* diazepam/clonazepam for epilepsy, or muscle spasms) or for off-label indications, appropriateness for deprescribing was assessed based on diagnosis, safety criteria and frequency of use.

Patients who reported ‘’I do not know’’ (IDK) when it comes to length of medication use were considered to be long-term users based on reported symptoms and diagnosis (*i.e.* reports chronic pain and IDK for when NSAID/OPIOID was started).

Reported *pro re nata* use was reviewed according to stated frequency and severity symptoms and diagnoses. For example, if a patient reports PRN use of a NSAID, chronic pain, level of pain and current or past diagnosis of pain disorders are reviewed. Patients reporting use of analgesics more than three times a week were considered to be scheduled users and not PRN users.

Adverse drug effects (ADE) were assessed based on reported frequency and severity of symptoms, changes in cognitive status, level of pain and pain control, and diagnosis. If a reported ADE could be contributed to more than one medication, the most likely correlation was chosen (*i.e.* dizziness with simultaneous use of BZN and an NSIAD). If a certain patient had more than one medication which could cause the ADE, additional annotation was given (*i.e.* patient reports leg oedema while using an NSIAD and Ca^2+^ channel blockers).

REFERENCES:

1. Farrell Pharmd B, Fcshp A, Pottie K, et al. Clinical Practice Guidelines Deprescribing proton pump inhibitors Evidence-based clinical practice guideline. *Can Fam Physician • Le Médecin Fam Can*. 2017;63.

2. Targownik LE, Fisher DA, Saini SD. AGA Clinical Practice Update on De-Prescribing of Proton Pump Inhibitors: Expert Review. *Gastroenterology*. 2022;162(4):1334-1342. doi:10.1053/j.gastro.2021.12.247

3. Katz PO, Dunbar KB, Schnoll-Sussman FH, Greer KB, Yadlapati R, Spechler SJ. ACG Clinical Guideline for the Diagnosis and Management of Gastroesophageal Reflux Disease. *Am J Gastroenterol*. 2022;117(1):27-56. doi:10.14309/AJG.0000000000001538

4. Kanno T, Moayyedi P. Who Needs Gastroprotection in 2020? *Curr Treat Options Gastroenterol*. 2020;18(4):557. doi:10.1007/S11938-020-00316-9

5. Abrignani MG, Gatta L, Gabrielli D, et al. Gastroprotection in patients on antiplatelet and/or anticoagulant therapy: a position paper of National Association of Hospital Cardiologists (ANMCO) and the Italian Association of Hospital Gastroenterologists and Endoscopists (AIGO). 2021;85:1-13. doi:10.1016/J.EJIM.2020.11.014

6. Primary health Tasmania A. *Non- Steroidal Anti-Inflamatory Drugs Recommended Deprescribing Strategy (NSAIDs)*.; 2019.

7. Rashid R, Chang C, Niu F, et al. Evaluation of a Pharmacist-Managed Nonsteroidal Anti-Inflammatory Drugs Deprescribing Program in an Integrated Health Care System. *J Manag care Spec Pharm*. 2020;26(7):918-924. doi:10.18553/JMCP.2020.26.7.918

8. Ali A, Arif AW, Bhan C, et al. Managing Chronic Pain in the Elderly: An Overview of the Recent Therapeutic Advancements. *Cureus*. 2018;10(9). doi:10.7759/CUREUS.3293

9. Kolasinski SL, Neogi T, Hochberg MC, et al. Foundation Guideline for the Management of Osteoarthritis of the Hand, Hip, and Knee. *Arthritis Rheumatol*. 2020;72(2):220-233. doi:10.1002/art.41142

10. Mann NK, Mathes T, Sönnichsen A, et al. Potentially Inadequate Medications in the Elderly: PRISCUS 2.0 First Update of the PRISCUS List. *Dtsch Arztebl Int*. 2023;120(1-2):3. doi:10.3238/ARZTEBL.M2022.0377

11. O’Mahony D, Cherubini A, Guiteras AR, et al. STOPP/START criteria for potentially inappropriate prescribing in older people: version 3. *Eur Geriatr Med*. 2023;14(4):625-632. doi:10.1007/S41999-023-00777-Y/TABLES/2

12. American Geriatrics Society Beers Criteria® Update Expert Panel. American Geriatrics Society 2023 updated AGS Beers Criteria® for potentially inappropriate medication use in older adults. *J Am Geriatr Soc*. Published online 4 May 2023. doi:10.1111/JGS.18372

13. Faculty of Pain Medicine RC of A. Opioids Aware:A structured approach to opioid prescribing (Long term prescribing). Accessed March 4, 2023. https://fpm.ac.uk/opioids-aware-structured-approach-opioid-prescribing/long-term-prescribing

14. Mathieson S, Maher CG, Ferreira GE, et al. Deprescribing Opioids in Chronic Non-cancer Pain: Systematic Review of Randomised Trials. *Drugs*. 2020;80(15):1563-1576.

15. NSW Therepautic Advisory Group A. *Deprescribing Guide for Regular Long-Term Opioid Analgesic Use in Older Adults*.; 2018.

16. Primary health Tasmania A. Resources: Medication management - deprescribing. Published 2022. Accessed June 13, 2023. https://www.primaryhealthtas.com.au/resources/deprescribing-resources/

17. Primary health Tasmania A. *Opioids Recommended Deprescribing Strategy*.; 2019.

18. Wang Y, Wilson DL, Fernandes D, et al. Deprescribing Strategies for Opioids and Benzodiazepines with Emphasis on Concurrent Use: A Scoping Review. *J Clin Med*. 2023;12(5):1788. doi:10.3390/JCM12051788/S1

19. Lavan AH, Gallagher P, Parsons C, et al. STOPPFrail (Screening Tool of Older Persons Prescriptions in Frail adults with limited life expectancy): consensus validation. *Age Ageing*. 2017;46(4):600-607.

20. Agency for Medicinal Products and Medical Devices of Croatia H. Medicinal products database for dug group N05B and N05C (benzodiazepine receptor agonists). Medicinal Products Database. Accessed February 26, 2023. https://www.halmed.hr/Lijekovi/Baza-lijekova/#rezultati

21. Lee JY, Farrell B, Holbrook AM. Deprescribing benzodiazepine receptor agonists taken for insomnia: a review and key messages from practice guidelines. *Pol Arch Intern Med*. 2019;129(1):43-49.

22. NSW Therapeutic Advisory Group A. *Deprescribing Gudie for Benzodiazepines and Z-Drugs*.; 2018.

23. Pottie K, Fcfp C, Thompson W, et al. Deprescribing benzodiazepine receptor agonists Evidence-based clinical practice guideline. 2018;64.

24. Primary health Tasmania A. *A Guide to Benzodiazepines Recommended Deprescribing Strategy*.; 2019.

25. Primary health Tasmania A. *A Guide to the Use of Benzodiazepines in Older Adults*.; 2016.

26. The Royal Australian College of Generl Practitioners R. Evidence-based guidance for benzodiazepines. Prescribing drugs of dependence in general practice. Accessed February 22, 2023. https://www.racgp.org.au/clinical-resources/clinical-guidelines/key-racgp-guidelines/view-all-racgp-guidelines/drugs-of-dependence/part-b/evidence-based-guidance-for-benzodiazepines

27. Rodríguez-Pérez A, Alfaro-Lara ER, Albiñana-Perez S, et al. Novel tool for deprescribing in chronic patients with multimorbidity: List of Evidence-Based Deprescribing for Chronic Patients criteria. *Geriatr Gerontol Int*. 2017;17(11):2200-2207. doi:10.1111/GGI.13062

28. Seppala LJ, Petrovic M, Ryg J, et al. STOPPFall (Screening Tool of Older Persons Prescriptions in older adults with high fall risk): a Delphi study by the EuGMS Task and Finish Group on Fall-Risk-Increasing Drugs. *Age Ageing*. 2021;50(4):1189-1199. doi:10.1093/AGEING/AFAA249

29. American Geriatrics Society. AGS Clinical Practice Guidelines and Recommendations. https://geriatricscareonline.org/ProductTypeStore/guidelines-recommendations-position-statements-/8/

30. British Geriatrics Society. Clinical guidelines. https://www.bgs.org.uk/resources/resource-series/clinical-guidelines

Deprescribing Potential of Commonly Used Medications among Community-Dwelling Older Adults: Insights from a Pharmacist-led Geriatric Assessment-APPENDIX FILE 2

Appendix table 1 Participants’ characteristics

| Characteristic | N=388 participants |
| --- | --- |
| age (median, IQR) | 73 years (IQR 69-79.75) |
| gender (female, n; %) | 247; 63.65% |
| region (n; %) |  |
| north-west continental  north-east continental  coastal | 144; 37.11%  125; 32.22%  119; 30.67% |
| number of medicines (median, IQR) | 6 (IQR 4-8) |
| number of diagnosis (median, IQR) | 5 (IQR 3-8) |
| last hospitalization (n; %of participants)^a^ |  |
| within the last 12 months  more than 12 months ago | 51; 13.86%  317; 86.14% |
| emergency department visits (n; % of participants)^a, b^ |  |
| yes  no | 97; 25.06%  290; 74.94% |
| utilization of other healthcare services (n; %of participants) ^a, b^ | |
| yes  no | 54; 14.14%  328; 85.86% |
| self-reported health status (n; %of participants)^a^ |  |
| very poor  poor  moderate  good  very good | 6; 1.55%  36; 9.39%  151; 39.02%  142; 36.69%  52; 13.44% |
| frailty score (n; %of participants) |  |
| non frail (score 3 or less)  frail (score 4 or higher) | 285; 74.22%  25.78% |
| length of medicine use (median, IQR)^c^ |  |
| PPI  NSAID  OPIOID  BZN | 4 years (IQR 2-6)  3 years (IQR 2-5 years)  2.5 years (IQR 2-5 years)  5 years (IQR 2-10 years) |
| type of medicine (n; %of participants) |  |
| use of single type of medicine  PPI  NSAID  OPIOID  BZN  combination of two medicines  PPI + NSAID  PPI + OPIOID  PPI + BZN  NSAID + OPIOID  NSAID + BZN  OPIOID + BZN  combination of three medicines  PPI + NSAID + OPIOID  PPI + NSAID + BZN  PPI + OPIOID + BZN  NSAID + OPIOID + BZN  combination of four medicines  PPI + NSAID + OPIOID + BZN | 31; 7.99%  46; 11.86%  13; 3.35%  46; 11.86%  **22.16%**  7; 1.80%  10; 2.58%  31; 8.25%  5; 1.29%  23; 5.92%  9; 2.32%  **11.33%**  3; 0.77%  24; 6.18%  13; 3.35%  4; 1.03%  3; 0.77% |
|  |  |

Appendix table 2 analysis of deprescribing criteria

| **CRITERIA** | **PPI** | | **NSAID** | | **OPI** | | **BZN** | | |
| --- | --- | --- | --- | --- | --- | --- | --- | --- | --- |
| **total number of deprescribing candidates**  **(n, % of users)** | 31.14% (n=38/122) | | 74.77% (n=83/111) | | 75.00% (n=45/60) | | 96.10% (n=148/154) | | |
| **LACK OF INDICATION**  **(n, % of users)** | 7.37% (n=9/122) | | none | | 18.33% (n=11/60) | | 24.67% (n=38/154)  15.03% (n=23/154) using 2 or more different BZN at the same time | | |
| **INAPPROPRIATELY LONG USE**  **(n, % of users)** | 26.23% (n=32/122) | | 52.25% (n=58/111) | | 70.00% (n=42/60) | | 61.04% (n=94/154) for insomnia use  47.71% (n=73/154) for anxiety use | | |
| **INAPPROPRIATE DOSE**  **(n, % of users)** | 12.29% (n=15/122) users had inappropriately high gastroprotective dose | | 17.12% (n=19/111) higher than recommended daily dose | | none | | 16.99% (n=26/154)  higher than recommended daily dose | | |
|  | 4.09% (n=5/122) prescribed inappropriate gastroprotective dose for NSAID users | |  |  |  |  |  |  |  |
| **SAFETY CONCERNS**  **(n, % of users)** | 2.46% (n=3/122) potential clinically significant DDI | | 40.54% (n=45/111) presence of adverse effects | | 56.33% (n=32/60)  presence of adverse effects | | 52.59% (n=81/154) presence of adverse effects | | |
|  |  |  | 30.97% (n=35/111) risk factors which could be exacerbated by NSAID use | | 51.67% (n=31/60) potential clinically significant DDI | | 36.60% (n=56/154) frailty score 4 and above | | |
|  |  |  | 32.43% (n=36/111) potential clinically significant DDI | |  |  | 25.94% (n=39/154) potential clinically significant DDI | | |
| **number of potentially clinically significant DDI (n, % )** | 3 | | 51 | | 50 | | 64 | | |
| **type of interaction and number** | X (esomeprazole-cefuroxime) | 1 | X (diclofenac-ibuprofen) | 4 | X (tramadol-carbamazepine) | 1 | X (alprazolam-clarithromycin) | | 1 |
|  | X (pantoprazole- cefuroxime) | 1 | X (ibuprofen-ketoprofen) | 3 | D (tramadol-bilastine) | 2 | D (alprazolam-zolpidem) | | 8 |
|  | X (pantoprazole-pazopanib) | 1 | X (indomethacin-naproxen) | 1 | D (tapentadol-lamotrigine) | 1 | D (diazepam-zolpidem) | | 6 |
|  |  |  | X (diclofenac-ketoprofen) | 1 | D (tapentadol-loratadine) | 1 | D (oxazepam-zolpidem) | | 3 |
|  |  |  | X (piroxicam-ketoprofen) | 1 | D (tramadol-moxonidine) | 3 | D (lorazepam-zolpidem) | | 1 |
|  |  |  | X (celecoxib-ibuprofen) | 1 | D (tramadol-promazine) | 1 | D (zolpidem-loratadine) | | 2 |
|  |  |  | D (ASA-ibuprofen) | 10 | D (tapentadol-pregabalin) | 1 | D (zolpidem-aripiprazole) | | 1 |
|  |  |  | D (ASA-ketoprofen) | 6 | D (tapentadol-quetiapine) | 1 | D (zolpidem-mirtazapine) | | 1 |
|  |  |  | D (ASA-diclofenac) | 3 | D (tramadol-sulpirid) | 1 | D (zolpidem-moxonidine) | | 1 |
|  |  |  | D (ASA-naproxen) | 2 |  |  | D (zolpidem-quetiapine) | | 1 |
|  |  |  | D (ASA-piroxicam) | 1 |  |  | D (zolpidem-trazodon) | | 1 |
|  |  |  | D (ibuprofen-sertraline) | 3 | D (alprazolam-tramadol) | | | | 13 |
|  |  |  | D (diclofenac-topical diclofenac) | 2 | D (diazepam-tramadol) | | | | 7 |
|  |  |  | D (ibuprofen-furosemid) | 2 | D (oxazepam-tramadol) | | | | 6 |
|  |  |  | D (naproxen-furosemide) | 1 | D (zolpidem-tramadol) | | | | 6 |
|  |  |  | D (diclofenac-escitalopram) | 1 | D (nitrazepam-tramadol) | | | | 3 |
|  |  |  | D (ibuprofen-escitalopram) | 1 | D (lorazepam-tramadol) | | | | 1 |
|  |  |  | D (piroxicam-indapamide) | 1 | D (zolpidem-tapentadol) | | | | 1 |
|  |  |  | D (diclofenac- hydrochlorothiazide) | 1 | D (diazepam-tapentadol) | | | | 1 |
|  |  |  | D (dexketoprofen-methotrexate) | 1 |  | | | |  |
|  |  |  | D (dexketoprofen-rivaroxaban) | 1 |  |  |  |  |  |
|  |  |  | D (dexketoprofen-rivaroxaban) | 1 |  |  |  |  |  |
|  |  |  | D (ibuprofen-rivaroxaban) | 1 |  |  |  |  |  |
|  |  |  | D (diclofenac-warfarin) | 1 |  |  |  |  |  |
|  |  |  | D (ibuprofen-warfarin) | 1 |  |  |  |  |  |
|  |  |  | D (naproxen-warfarin) | 1 |  |  |  |  |  |
| **additional information** | 4.09% (n=5/122) reported PRN use | | 18.92% (n=21/111) reported PRN use | | 23.33% (n=14/60) reported PRN use | | | 26.80% (n=41/154) reported PRN use | |
|  | 50.00% (n=61/122) of users reported GI symptoms  17.21% (n=21/122)  needs PPI for gastroprotection but had it prescribed for other diagnosis | | In 6.30% (n=7/111) users adverse effects could be associated with use of other medicines | | In 30.00% (n=18/60) users adverse effects could be associated with use of other medicines | | | In 35.71% (n=55/154) adverse effects could be associated with use of other medicines | |

*PPI- proton pump inhibitors, NSAID- nonsteroidal anti-inflammatory drugs, OPI-opioid analgesics, BZN- benzodiazepine receptor agonists, DDI- drug-drug interaction, ADE- adverse drug effects, PRN- pro re nata use, ASA- acetylsalicylic acid*
